# Cellular dynamics as a marker of normal-to-cancer transition in human cells

**DOI:** 10.1038/s41598-023-47649-w

**Published:** 2023-11-29

**Authors:** M. P. M. Marques, A. L. M. Batista de Carvalho, C. B. Martins, J. D. Silva, M. Sarter, V. García Sakai, J. R. Stewart, L. A. E. Batista de Carvalho

**Affiliations:** 1 Molecular Physical-Chemistry R&D Unit, Department of Chemistry, University of Coimbra, 3004-535 Coimbra, Portugal; 2 Department of Life Sciences, University of Coimbra, 3000-456 Coimbra, Portugal; 3 STFC Rutherford Appleton Laboratory, ISIS Facility, Chilton, Didcot, OX11 0QX UK

**Keywords:** Biochemistry, Biophysics, Cancer

## Abstract

Normal-to-cancer (NTC) transition is known to be closely associated to cell´s biomechanical properties which are dependent on the dynamics of the intracellular medium. This study probes different human cancer cells (breast, prostate and lung), concomitantly to their healthy counterparts, aiming at characterising the dynamical profile of water in distinct cellular locations, for each type of cell, and how it changes between normal and cancer states. An increased plasticity of the cytomatrix is observed upon normal-to-malignant transformation, the lung carcinoma cells displaying the highest flexibility followed by prostate and breast cancers. Also, lung cells show a distinct behaviour relative to breast and prostate, with a higher influence from hydration water motions and localised fast rotations upon NTC transformation. Quasielastic neutron scattering techniques allowed to accurately distinguish the different dynamical processes taking place within these highly heterogeneous cellular systems. The results thus obtained suggest that intracellular water dynamics may be regarded as a specific reporter of the cellular conditions—either healthy or malignant.

## Introduction

Cancer is a worldwide health problem and the second leading cause of death globally—almost 10 million deaths in 2020 (from 19.3 million new cases), expected to rise up to 28.4 million cases in 2040 (a 47% rise in two decades)^1^. Female breast carcinoma is currently the most commonly diagnosed type of cancer (11.7%), followed by lung (11.4%), colorectal (10.0%) prostate (7.3%) and stomach (5.6%) cancers. Triple-negative breast adenocarcinoma, in particular, is the most aggressive type of mammary cancer with a prevalence in younger women and a very poor prognosis, since little therapeutic progress has been achieved in the last decades^2^. Lung cancer is the second most common type of neoplasia, with a very low survival rate (10–20% 5-year survival after diagnosis) and the leading cause of global cancer mortality (18.0% of the total cancer deaths), mainly due to its late diagnosis (only 16% of lung cancer cases are diagnosed early). Prostate carcinoma, in turn, is the most common cancer in men in western countries, the metastatic androgen-independent type being currently incurable^3,4^. The heterogeneity of human malignancies and the poor knowledge of normal-to-cancer (NTC) transformation, at a molecular an functional levels, pose a challenge to the development of effective diagnosis and therapeutic strategies.

A sustained disruption of cellular homeostasis is known to trigger a cascade of events that induce the transformation of one cell type into another, namely during carcinogenesis and malignant progression^5^. This transition from normal-to-cancer state is a complex non-equilibrium phenomenon still very poorly understood, despite its undisputable impact on human health. Apart from the biochemical hallmarks of cancer metabolism^6–9^, carcinogenesis is recognised to be intimately related to the cell´s biomechanical profile, in particular to intracellular water dynamics^10–12^. Since water provides the matrix in which all biochemical processes occur, its integrity (structural and dynamical) is fundamental for maintaining a healthy cellular state. In the highly crowded cytoplasm water displays particular properties, different from those of bulk water, and plays a fundamental role in normal cell activity: through maintenance of the 3D architecture and functional conformation of biopolymers (via their hydration layers) and by regulating vital biological processes (e.g. protein synthesis, energy generation and cellular signalling)^13–16^. Any alterations in the properties of cellular water can be the driving force to disrupt homeostasis and initiate a series of events leading to biomolecular disfunction, that can facilitate neoplastic growth and progression^17–21^. Hence, elucidating water dynamical behaviour in both tumourigenic and non-tumourigenic states is crucial for unravelling the NTC transition as well as the progression from localised to metastatic tumours.

Quasielastic neutron scattering (QENS) measures very small energy transfers which occur upon an inelastic interaction between a neutron beam and the sample, providing information on diffusive motions within the system at the sub-nanosecond timescale and nanometer lengthscale, being particularly sensitive to hydrogen atoms. Hence, it is a highly suitable experimental approach for accessing the low-energy vibrations of biomolecules and studying hydrogen-atom dynamics in biological systems, namely regarding water confined in tissues or cells (either within the cytoplasm or hydration layers)^22–35^. QENS allows to directly probe different spatially resolved dynamical processes—from fast localised modes to slower global motions. Former QENS studies by the authors have evidenced the importance of this technique to accurately monitor cytoplasmic and hydration water dynamics in both whole cells and hydrated biomolecules (e.g. DNA), particularly concerning: (i) the impact of anticancer drugs on the behaviour of interfacial water^27,36–38^; (ii) the role of water dynamics in normal-to-cancer transition^34,35,39^. Regarding the latter, the results pointed to a significantly higher plasticity for cancerous cells, this enhanced mobility of intracellular water being a potential hallmark of malignancy. Furthermore, the histological nature of the tumour was found to be associated with specific dynamical profiles. Building on the success of these experiments, in particular the studies of human cancer cells versus their non-tumourigenic counterparts^39^, the present work aims at extending this approach to other types of cells and at different incident neutron energies, with a view to achieve a comprehensive understanding of the variations in cellular dynamics underlying oncogenic transformation and tumour aggressiveness, in a wide range of malignancies. This is paramount for developing better diagnosis and chemotherapeutic approaches, aiming at an improved prognosis for oncological patients.

The cold neutron spectrometer LET at the ISIS Neutron Source (UK)^40^ is particularly well suited to probe water dynamics in biological systems, since with multi-repetition rate multiplication it can access different dynamical processes in one measurement, spanning multiple time and length scales, with the advantage of delivering different energy windows in one measurement (three incident neutron energies). This is key to understanding the highly heterogeneous cellular samples currently under investigation, which have been shown to display several types of motions—from diffusion of intracellular water (both in the cytoplasm and in biomolecules´ hydration layers) to fast internal localised dynamics^34,39^. The goal of the current study was therefore to use QENS in a wide energy range to monitor healthy and cancerous human cells (from breast, prostate and lung), as an innovative approach for unveiling particular QENS signatures associated to normal-to-cancer transition and identifying specific reporters of cancer.

## Materials and methods

The list of chemicals and the detailed experimental protocol for the preparation of the cell samples are described in the Supplementary Material, as well as specific procedures regarding the QENS measurements and analysis.

### Cell samples

Three human cancer cell lines were studied, along with their non-tumourigenic (healthy) equivalents (Table 1): (i) triple-negative breast carcinoma, breast cancer (MDA-MB-231) and non-transformed mammary cells, breast healthy (MCF-12A); (ii) androgen-independent prostatic adenocarcinoma, prostate cancer (PC-3) and normal prostate epithelial cells, prostate healthy (PNT-2); (iii) lung cancer (A549) and normal bronchial epithelium cells, lung healthy (BEAS-2B). The cells were cultured on-site (at the Biology laboratory of the ISIS Pulsed Neutron and Muon Source of the Rutherford Appleton Laboratory), following the protocol used for previous experiments^37,39^, yielding at least 150 mg of pellet per sample (1 cm^3^, containing *ca.* 5 × 10^8^ cells) (see details in Supplementary Material).

**Table 1:** Designation used along the text for the human cells lines studied in the present work, and their ratio (intracellular water mass): (biomass).

Cell line	Designation	(Intracellular water mass): (biomass) (%)
MDA-MB-231	Breast cancer	20.2
MCF-12A	Breast healthy	19.1
PC-3	Prostate cancer	24.2
PNT-2	Prostate healthy	19.5
A549	Lung cancer	14.2
BEAS-2B	Lung healthy	13.1

Prior to the QENS measurements, the cell pellets were washed with deuterated phosphate buffered saline (PBS_deut_) and centrifuged in order to remove the extracellular water, following a previously optimised procedure^37,39^ (see Supplementary Material). These experimental conditions ensure that, for all types of cells, extracellular water is always below 5% of the total water present, all the remaining being intracellular water (95%) (the contribution of extracellular water to the scattering profile thus being negligible).

The ratio (intracellular water mass): (biomass) (representing the weight of intracellular water *vs* the weight of the lyophilised cell pellet), which is expected to differ for each type of cell, was determined: breast healthy—19.1%; breast cancer—20.2%; prostate healthy—19.5%; prostate cancer—24.2%; lung healthy—13.1%; lung cancer—14.2% (Table 1).

### QENS measurements

The QENS experiments were performed at the ISIS Pulsed Neutron and Muon Source of the Rutherford Appleton Laboratory^40^, in the low-energy LET direct-geometry time-of-flight spectrometer^41,42^, which allows to probe water dynamics at picosecond timescales and on atomic lengths, over a wide energy range and with multiple incident neutron energies in the same measurement (1.7, 2.97 and 6.42 meV, see details in the Supplementary Material).

All samples were analysed (in flat Al cans) at 310 K, to better represent physiological conditions. Data for the deuterated saline buffer (PBS_deut_) was also obtained, for comparison purposes. The scattering signal from an empty flat Al can was taken as the background.

The QENS data was reduced from raw time-of-flight signals into energy transfer spectra using the MANTID package (version 6.5.0)^43^. Fitting of the QENS spectra was performed with the program DAVE (version 2.5, National Institute of Standards and Technology (NIST) Center for Neutron Research, USA) ^44^ (see details in the Supplementary Material).

## Results and discussion

The QENS scattering profiles measured for the cell pellets washed with deuterated saline buffer solution are dominated by contributions from intracellular water, as well as from cellular metabolites and biomolecules. The LET spectrometer at ISIS, with three energy windows in one measurement, allows us to detect different dynamical processes with high accuracy. This is particularly helpful in highly heterogeneous samples such as cells which comprise multiple dynamics to be interpreted, from slow motions (e.g. water confined in hydration layers) to fast dynamical contributions (e.g. water rotations or internal localised motions within biomolecules). Fast diffusion processes lead to a broad energy distribution of the scattered neutron beam, yielding quasi-elastic curves with a large full width at half-maximum (FWHM), while slow motions generate narrower QENS profiles. Due to the wavelength-dependent source flux, the QENS signal at the lowest incident neutron energy is significantly weaker.

**Figure 1:**
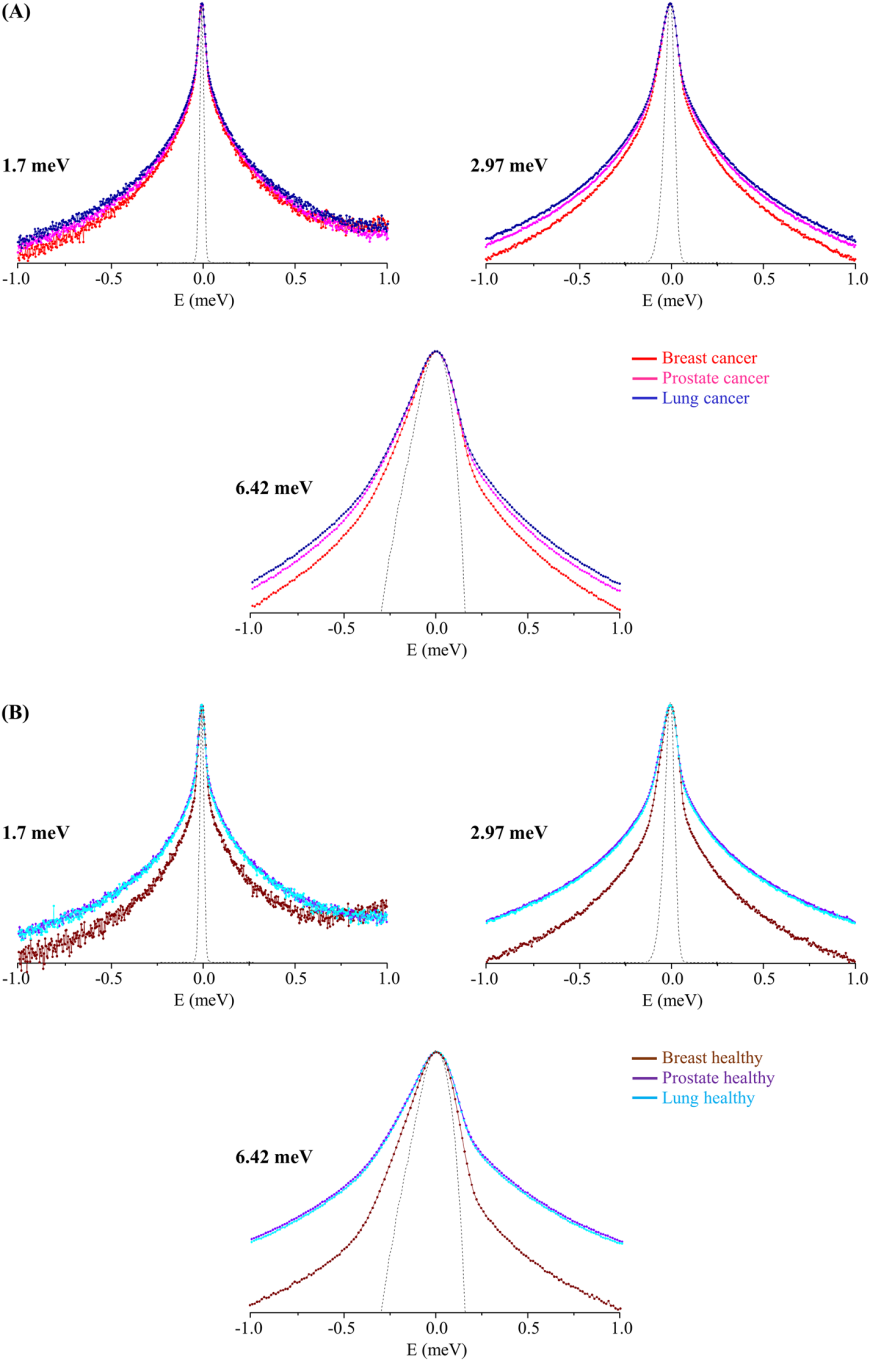
QENS profiles (at 310 K, summed over all Q values, at 1.7, 2.97 and 6.42 meV incident energies) for all cells under study—breast, prostate and lung: cancer (**A**) and healthy (**B**). (The spectra were normalised to the maximum peak intensity. The dashed line represents the instrument resolution, as measured by a standard vanadium sample. The QENS profiles are represented in *yy′* logarithmic scale).

Attending to the different chemical profiles (e.g. regarding protein or lipid composition) and varying biomechanical features of distinct types of tumours^45–47^, three human cancer cells were probed—breast, prostate and lung—along with their non-tumourigenic equivalents. The QENS profiles obtained for the malignant cells clearly evidence that the lung cells display a higher flexibility relative to prostate and breast cancers, the latter being the least mobile (Fig. 1A). This trend is followed by the corresponding healthy cells only for breast, since healthy prostate and lung display very similar QENS profiles (Fig. 1B). Hence, while for malignant cells the differences in dynamical behaviour are visible for the three types of cells, this being more distinctive for the faster dynamical contributions (better observed at 2.97 and 6.42 meV incident energies), for the non-malignant systems only breast cells stand out as the least flexible. In addition, the differences in dynamical profile are considerable larger between healthy cells as compared to cancer, being already observed at 1.7 meV incident energy (slowest motions).

**Figure 2:**
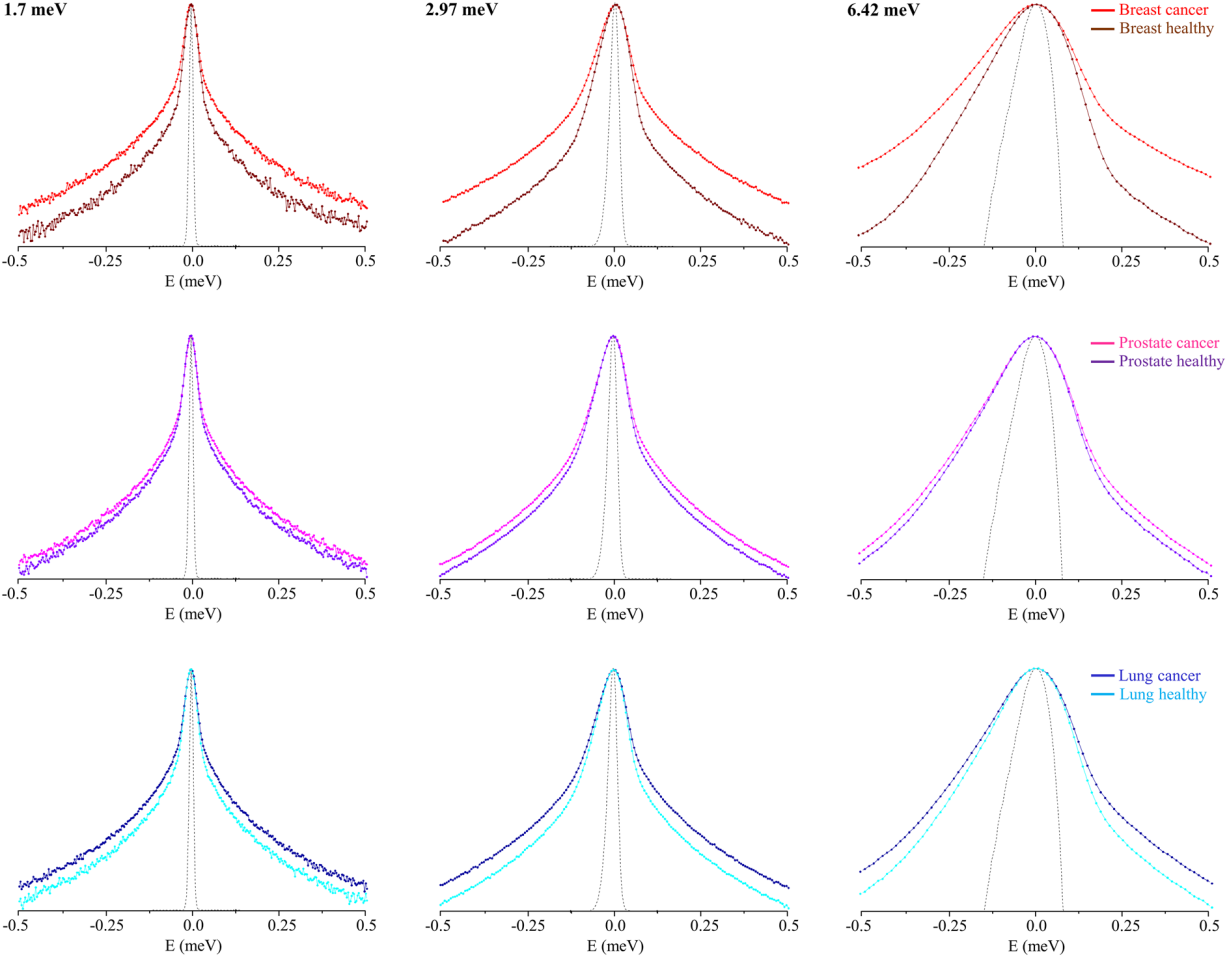
QENS profiles (at 310 K, summed over all Q values, at 1.7, 2.97 and 6.42 meV incident energies) for all cancer cells under study versus their healthy counterparts—breast, prostate and lung. (The spectra were normalised to the maximum peak intensity. The dashed line represents the instrument resolution, as measured by a standard vanadium sample. The QENS profiles are represented in *yy´* logarithmic scale).

When comparing tumour and healthy cells, the difference in dynamics is more significant for breast cells when compared to prostate and lung (Fig. 2), as previously reported for the former two types of cells^39^ based on QENS data measured in the OSIRIS instrument (at identical resolution but only one incident energy). Actually, breast cells display the largest variation in dynamics upon normal-to-malignant transition, this difference being more marked for the faster dynamical components (data obtained with higher accuracy at the 2.97 and 6.42 meV incident energies). In turn, prostate cells show the smallest dynamical changes between healthy and cancer states. Several studies have evidenced significant differences in intracellular water between healthy and cancerous cells, a clear correlation between malignancy and cellular plasticity having been revealed—tumour cells displaying a higher flexibility as compared to healthy ones, this enhanced deformability being even more noteworthy for metastatic malignancies^11,17,48–52^. These particular biomechanical properties are suggested to allow cancer cells to grow uncontrollably, adapt to hostile microenvironments, and acquire the ability to become invasive and metastasize.

It should be emphasised that the different values of the (intracellular water mass): (biomass) for the three distinct cell lines currently probed, both healthy and cancer (breast, prostate and lung, Table 1), are not expected to affect the interpretation of the QENS results, since this is solely based on the dynamical behaviour of intracellular water—the same for all cellular systems (*ca.* 95%). Moreover, although this ratio differs according to the type of cell the water´s molar fraction is so much larger relative to all other cellular components that those differences should not interfere with the QENS results.

An accurate fitting of the experimental data is key for a reliable interpretation of the results based on a precise identification and discrimination of the several dynamical processes occurring within the cellular millieu. Presently, fitting was achieved with one Delta function (elastic component) convoluted with the lineshape of the instrument and Lorentzian functions to represent the quasielastic contributions, following the model previously optimised by the authors and applied to breast, prostate and bone carcinoma cells^27,37,39^. This model has also been used successfully by other authors to represent microscopic diffusion processes in living planarians^53^. Attending to the high degree of complexity of the heterogeneous biological matrices under study, three Lorentzian functions were found to be needed for a full fitting of the data: beyond Γ_local_ to characterise the fast localised dynamics of the biomolecules (e.g. DNA, proteins and lipids, which cannot be discriminated in the timescale of LET) and the rotation of cytosolic water, two other Lorentzians are required to represent the intracellular water molecules (Γ_global_), which have distinct dynamical regimes depending on their location—either in the cytoplasm (higher mobility, Γ_global/cyt_) or in the more restricted hydration layers surrounding biomolecules (Γ_global/hyd_). Three major dynamical contributions were therefore considered (Fig. S1, Supplementary Material): (i) very slow motions from the biomass alone (excluding water), slower than the longest observable time defined by the spectrometer resolution—largest organelles, cytoskeleton and global motions of the macromolecules—represented by a Delta function; (ii) slow diffusion of the intracellular water molecules (Q-dependent reorientations mediated by hydrogen bonds)—cytoplasmic and hydration water—defined by two different Lorentzian functions (Γ_global_); (iii) internal localised motions (Q-independent)—biomolecules´ conformational rearrangements and lipid motions (e.g. lateral diffusion of phospholipids and cholesterol diffusion across membranes)—ascribed to a broader Lorentzian (Γ_local_). Based on this procedure, a viable representation was attained which enabled us to unveil the dependence of the QENS peak width (FWHM = Γ) of the Lorentzian functions (representing the cellular dynamical contributions) on the transferred momentum (Q^2^), and relate each diffusional motion with a length scale and a dynamical model. Previous analysis of data measured for several human cancer and non-malignant cells (also fitted with three convoluted Lorentzian functions) allowed to identify three processes of the order 0–0.1 meV, 0.2–0.8 meV and 2–3 meV^39^. LET, at a setting that can access these three energy ranges, enabled us to differentiate and much more accurately assign each of these three processes in one go. The data currently measured at a 1.7 meV incident energy represents the slowest dynamical component in the cellular systems (hydration water motions), while the results acquired at 2.97 and 6.42 meV correspond, respectively, to the water dynamics within the cytoplasm, and to fast water rotations and internal motions in biomolecules.

**Figure 3:**
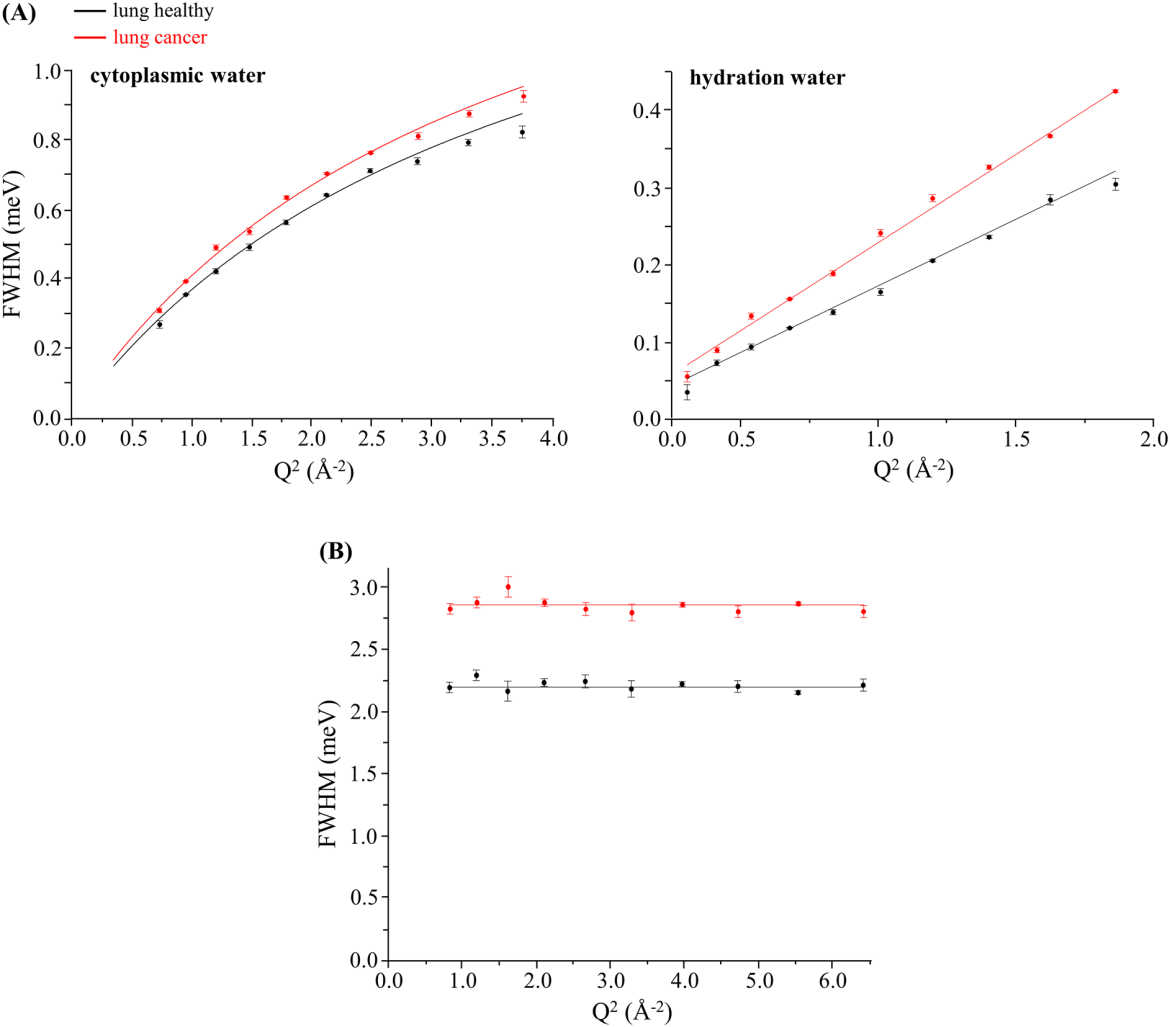
Variation of the full widths at half-maximum (FWHM) with Q^2^ for human lung cancer and lung healthy cells (at 310 K): (**A**) Lorentzian functions representing the translational motions of intracellular water—cytoplasmic medium and hydration layers (Γ_global_) (data measured at 2.97 and 1.70 meV incident neutron energies, respectively); (**B**) Lorentzian function representing the internal localised motions within the cell (Γ_local_) (data measured at a 6.42 meV incident neutron energy).

It has been shown^27,33–35^ that water in biospecimens (such as cells and tissues) does not always display a simple diffusion behaviour (Fickian diffusion), but, instead, its motions may be restricted by strong intermolecular interactions (e.g. H-bonds) which results in jumps of the water molecules from one site to another (jump-diffusion), with a specific diffusion coefficient and residence time (τ) between diffusion steps. Thus, for a quantitative interpretation of the QENS results the data was fitted to either a jump-diffusion model or a Fickian model over the Q-range measured (Fig. 3), yielding the quantitative parameters—diffusion coefficients (D_T_) and residence times (τ_T_)—characterising the various dynamical contributions (Table 2). While the cytoplasmic and hydration water dynamics exhibit a Q-dependent behaviour (Fig. 3A), the fast localised motions show a Q-independent profile (Fig. 3B). In addition, the flexibility of the water molecules in the cytoplasm was found to be larger (as expected) than within the highly ordered hydration layers—the former following a non-diffusive jump reorientation behaviour^54–56^ (Γ_global_ increasing asymptotically to a plateau, Eq. (5) of the Supplementary Material), while the latter obeys to a Fickian diffusion model (Fig. 3A). This noteworthy dynamical profile of water in hydration shells was previously observed for breast and prostate cells^39^, and is in contrast with results reported for these same types of cells upon exposure to a drug which displayed a non-Fickian dynamical profile (due to the drug impact on intracellular water dynamics)^27,37^.

**Table 2:** Translational diffusion coefficients (D_T_) and relaxation times (τ_T_, τ_L_) of intracellular water (at 310 K) for cancer and non-cancer human cells: breast cancer *vs* breast healthy; prostate cancer *vs* prostate healthy; lung cancer *vs* lung healthy.

Cell line	Γ_global/cyt_		Γ_global/hyd_	Γ_local_
	D_T_ (× 10^–5^ cm^2^ s^−1^)	τ_T_ (ps)	D_T_ (× 10^–5^ cm^2^ s^−1^)^b^	τ_L_ (ps)
Breast cancer	0.73 ± 0.01	0.42 ± 0.01	0.09 ± 0.01	0.59 ± 0.01
	1.13 ± 0.05^a^	0.60 ± 0.02^a^	0.17 ± 0.01^a^	0.56 ± 0.01^a^
Breast healthy	0.58 ± 0.01	0.46 ± 0.01	0.05 ± 0.01	0.60 ± 0.01
	^*a*^1.01 ± 0.06	^*a*^0.60 ± 0.03	^*a*^0.15 ± 0.02	^*a*^0.59 ± 0.01
Prostate cancer	0.74 ± 0.02	0.37 ± 0.02	0.10 ± 0.01	0.37 ± 0.01
	1.31 ± 0.01^a^	0.56 ± 0.01^a^	0.21 ± 0.02^a^	0.36 ± 0.04^a^
Prostate healthy	0.61 ± 0.02	0.38 ± 0.02	0.08 ± 0.01	0.49 ± 0.01
	1.25 ± 0.04^a^	0.57 ± 0.01^a^	0.18 ± 0.01^a^	0.48 ± 0.01^a^
Lung cancer	0.80 ± 0.02	0.36 ± 0.02	0.12 ± 0.01	0.35 ± 0.01
Lung healthy	0.71 ± 0.02	0.38 ± 0.02	0.09 ± 0	0.45 ± 0.01

Slow global translational (Γ_global/cyt_ and Γ_global/hyd_) and fast localised (Γ_local_) dynamical processes.

^a^From^39^. ^b^Fickian behaviour (Γ = 2DQ^2^).

The diffusion coefficients (D_T_) and residence times (τ_T_) presently obtained (Table 2) clearly evidence particular dynamical features of tumour and non-tumour cells (in the picosecond timeframe) which allow to distinguish between them: a higher flexibility of the cytomatrix in malignant states as compared to healthy ones is confirmed by an increase in D_T_ values (*ca.* 26%, 20% and 12% respectively in breast, prostate and lung cancers), coupled to a decrease in τ_T_ (*ca.* 11%, 2% and 6% respectively in breast, prostate and lung cancers). This is accompanied by a similar trend for water motions in the hydration layers of cellular biomolecules, which show a very significant increased mobility upon malignant transformation (*ca.* 57%, 33% and 33% respectively in breast, prostate and lung cancers). Regarding the residence times for the faster localised motions of the cellular components (Γ_local_) a decrease upon malignant transition was found, reflecting a faster dynamics, which is particularly noteworthy for prostate and lung carcinomas—*ca.* 24% and 23%, respectively, as compared to *ca.* 2% for breast cancer.

**Figure 4:**
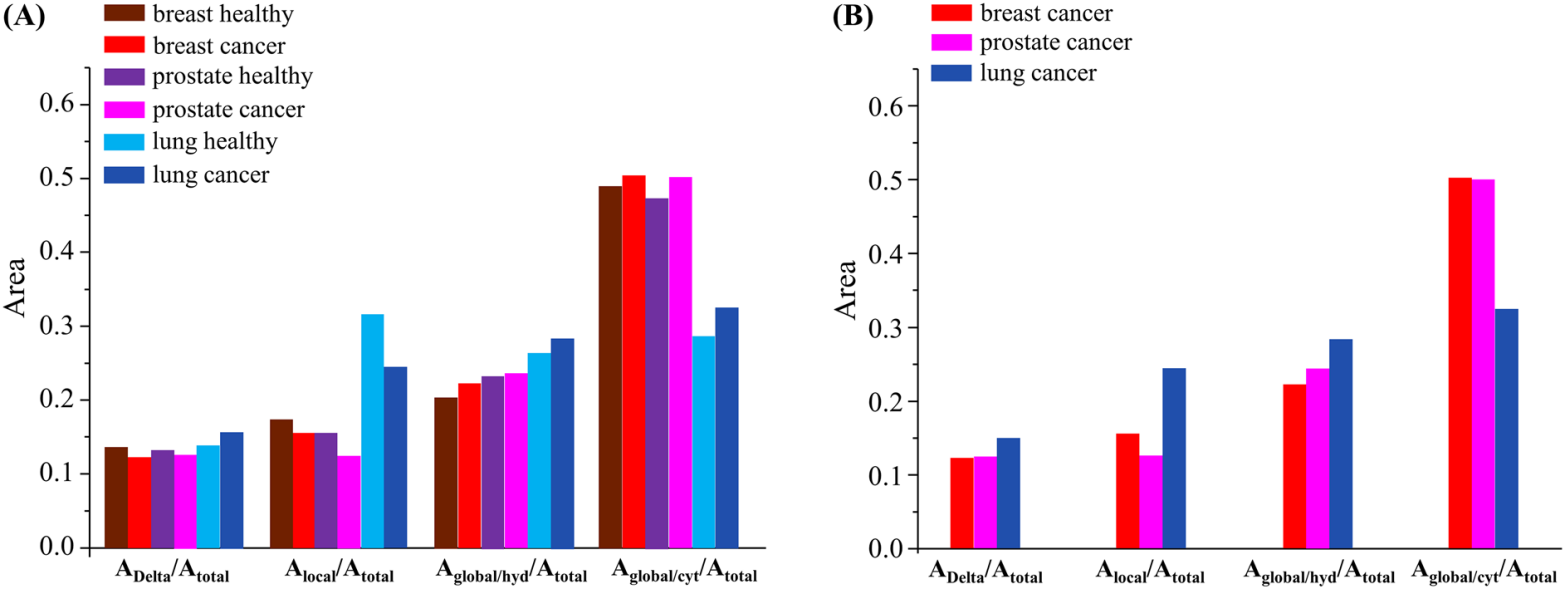
Area ratios for the different functions that characterise the dynamical behaviour of intracellular water (at 310 K) in the human cells under study, for Q = 1.217 Å^−1^ at an incident neutron energy of 2.97 meV—Delta function representing the slowest motions, outside the LET timeframe (A_Delta_); Lorentzian functions representing the localized rotations (A_local_) and the translational motions of cytoplasmic and hydration intracellular water (A_global/cyt_ and A_global/hyd_): (**A**) cancer *vs* healthy cells; (**B**) all cancer cells. (The data are plotted at a representative intermediate Q-value (1.217 Å^−1^); however, the trend was found to be similar across the bank).

The amplitudes of the different components used to characterise the dynamical profile of intracellular water are represented in Fig. 4, for the cancer and healthy cells under study: A_Delta_, for to the slowest motions (that may be considered as immobile components in the LET timeframe); A_global/cyt_ and A_global/hyd_, for the translations of cytoplasmic and hydration waters, respectively; A_local_, for the faster localised rotations; and A_total_, for the sum of the amplitudes of all dynamical contributions. These plots evidence the influence of immobile *versus* mobile species in the malignant and healthy cells, and highlight the relationship between NTC transformation and the distinct motions occurring within the cellular systems. Regarding the slowest motions (A_Delta_) their contribution is slightly lower in cancer *versus* healthy breast and prostate cells, within a quite narrow variation range, while it is higher for lung cancer *vs* lung healthy (Fig. 4A). This seems to suggest that the biochemical profile reflected by this immobile-to-mobile ratio (A_Delta_/A_total_) is similar for breast and prostate matrices, as opposed to lung. As for the dynamical processes that take place within these cells, clear mobility differences are observed between all malignant and non-malignant samples for both cytoplasmic and hydration water, evidencing a contribution for NTC transition from the translational motions of these types of water: both Γ_global/cyt_ and Γ_global/hyd_ are found to be higher in tumour samples (with the exception of Γ_global/hyd_ for prostate, which is virtually unchanged). There is a noteworthy smaller overall influence of Γ_global/cyt_ in lung cells (A_global/cyt_/A_total_ (at Q = 1.217 Å^−1^) equal to 0.3247 for lung *vs* 0.5022 and 0.5028 for breast and prostate respectively), which is in accordance with the corresponding D_T_ values that display a less marked increase in malignant *vs* healthy states for lung as compared to breast and prostate (*ca.* 26%, 20% and 12% respectively, Table 2). Finally, the local dynamics (Γ_local_) decreases for all malignant cells, with a major overall impact for lung (A_local_/A_total_ (at Q = 1.217 Å^−1^) equal to 0.2435 for lung cancer *vs* 0.1547 and 0.1273 for breast and prostate cancers respectively). This A_local_/A_total_ relationship follows the values of the (intracellular water mass): (biomass) ratios currently determined for the different cells under study (Table 1), this ratio increasing for the malignant *vs* the corresponding healthy cells, this rise being larger for prostate cells (1.1%, 4.7% and 1.1%, respectively for breast, prostate and lung cells). These results evidence a somewhat distinct dynamical behaviour from lung cancer relative to breast and prostate cells, namely regarding the enhanced contributions of translational motions from hydration water and localised fast rotations when going from healthy to tumourigenic states. When comparing the three types of cancer cells under study, the impact of the local (fast) motions and of the hydration water translations is clearly more significant for lung, while the contribution from cytoplasmic water dynamics is less important for this type of malignancy relative to breast and prostate (for which this dynamical component is noteworthy) (Fig. 4B).

The present results clearly show that the dynamical behaviour of intracellular water varies according to the nature of the tissue were the cancer originates (i.e. the cancer histology). From the breast, prostate and lung carcinomas under study, lung was shown to display the highest plasticity followed by prostate and metastatic breast (Fig. 1A). Actually, while the cancers currently probed are all from epithelial origin, malignancies from bone/connective tissue such as osteosarcoma were previously reported by the authors to have a remarkably lower overall flexibility^37^. In addition, the greater plasticity of neoplastic *versus* healthy cells has been proposed to be directly associated with their metastatic potential^10,20,46,51^, which was also verified by the current results—lung cancer being the most invasive one among the three types of malignancies currently investigated. Notably, these biomechanical differences are accompanied by biochemical variations recognised to be linked to an enhanced cell proliferation and motility^20,46,51,57,58^, as reported by the authors for several human cells and tissues, both cancer and healthy, through combined spectroscopic approaches [Raman, Fourier Transform Infrared (FTIR) and Nuclear Magnetic Resonance (NMR)]^7,8,37,59–61^. As an example, the presently probed metastatic breast cancer (triple-negative, lacking the oestrogen, progesterone and human epidermal growth factor receptors) displays a modified fatty acid profile, including an increased unsaturation degree, that may partly justify its increased plasticity^45,46^. Regarding lung cancer, in turn, apart from the dysregulated glucose metabolism (and consequent increased lactate and decreased pyruvate, Warburg effect), cell-free DNA (DNA released from the cells into the circulatory system) has been found in increased amounts in liquid biopsies^62,63^.

The current data can be compared with previous results obtained in the OSIRIS instrument^37,39^ (Table 2), since LET probes similar timescales to OSIRIS and the LET resolution at 1.7 meV incident energy (27 µeV) is identical to that of OSIRIS (25.4 µeV at a 1.84 meV incident energy for a PG002 25 Hz configuration). The D_T_ and τ_T_ values presently obtained for Γ_global/hyd_ (from the data measured at a 1.7 meV incident energy) are found to be lower than the formerly reported ones^39^, but they remain of the same order of magnitude and follow the same trend, consistent between the two instruments, regarding healthy *vs* cancer samples (Table 2). The discrepancies in the absolute values for these parameters may have a twofold justification: (i) although the same experimental protocol was followed, the cell pellets were newly prepared for the current experiment; (ii) QENS acquisition with three simultaneous incident energies—three matching datasets—yields much more accurate data than that previously gathered (at OSIRIS). The larger difference in dynamics between tumour and healthy cells for breast samples relative to prostate and lung is also in accordance with previous QENS data for the former two cell lines^39^. Furthermore, the present results agree with data found in the literature that compares the biomechanical properties of tumour and healthy cells: (i) reported Atomic Force Microscopy (AFM) experiments on prostate cancer cells (PC-3 cell line) evidenced a clear discrimination between malignant and non-malignant states based on their elastic properties, as well as between cancer cells at different stages of disease progression^48^. (ii) AFM studies in fluids from patients with lung and breast cancer showed that malignant metastatic cells were *ca.* 70% softer than their benign counterparts^10^.

## Conclusions

Effective tools for cancer diagnosis (including early diagnosis) rely on the discovery of cancer biomarkers, i.e. specific changes (biochemical and/or biomechanical) directly associated to NTC transformation, which can therefore help to distinguish between normal and cancerous conditions. However, such biomarkers are scarce, mainly for certain types of malignancies, since the mechanisms underlying tumour initiation, progression and metastasis are still poorly understood. Regarding the highly invasive and rapidly metastasizing lung cancer, in particular, there are still no biomarkers for an accurate detection in clinical use, which hinders an effective (and early) diagnosis. This is therefore an urgent clinical need, since the majority of these cancers are detected at a late stage precluding a successful treatment and contributing to a very high mortality rate.

Up to this date, several studies have reported a decrease in stiffness (increased plasticity) of human cells associated with cancer and metastatic ability, which has demonstrated the potential of cell´s dynamical properties as biomarkers of malignancy, and nanomechanical measurements as a promising tool for cancer detection (as a complement to the often challenging morphological analysis). Hence, the present study aims to contribute to a better understanding of the molecular basis of NTC transition, by probing intracellular dynamics through QENS, in healthy and cancer human cells from breast, prostate and lung, with particular emphasis on the motions of water (in the different cellular locations). The dynamical profiles obtained for cancer versus non-malignant cells showed a clear discrimination between the various dynamical components within the heterogenous cellular matrices. An increased motility is observed upon normal-to-malignant transformation, the lung carcinoma cells displaying the highest plasticity followed by prostate and breast cancer. The water dynamics, both within the cytoplasm and the biomolecules’ hydration layers, is shown to change from healthy to malignant specimens with a major contribution from the latter. Furthermore, lung cells show a distinct behaviour relative to breast and prostate, particularly regarding an enhanced contribution from hydration water motions and localised fast rotations upon NTC transition. These results corroborate the link between metabolism and cell´s biomechanical features, and allow us to conclude that intracellular water dynamics may be regarded as a specific reporter of the cellular state—healthy versus cancer.

Moreover, it should be emphasised that the biophysical description of malignancy currently retrieved from QENS is in accordance with the well-recognised biomechanical and biochemical differences reported for distinct types of cells, namely those currently under study. A very high heterogeneity and plasticity (as determined by methods such as cell biology and atomic force microscopy) have been reported as hallmarks of lung cancer^64^, justifying its prominent metastatic capacity which is responsible for a low survival rate. As for the breast malignancies, cell migration and angiogenesis (neovascularisation) experiments carried out by the authors on the most aggressive subtype^65^, the presently probed triple-negative breast cancer (MDA-MB-231 cell line), have shown a considerable invasive ability which is in accordance with other reports^66^ and with its clinically recognised metastatic capacity. Prostate cancer, in turn, is known to display a significant lineage plasticity (i.e. cellular differentiation and reprogramming), which is linked to drug resistance (as in the low prognosis castration-resistant prostate carcinoma)^67^. Overall, cellular function and biomechanics are deeply intertwined^68,69^, and metastasising tumour cells have recently been shown to display dynamic changes in order to survive in their variable microenvironment during the metastatic cascade (over 2/3 of metastatic cells exhibit a lower stiffness)^10,70^.

This mechano-metabolic crosstalk, and the correlation between QENS dynamical data and results on cell proliferation, plasticity and migration ability are invaluable for better understanding crucial processes such as abnormal cell growth (triggering displasia), NTC transformation, changes in tumour cell state, and invasive ability leading to metastatic establishment of cancers beyond the primary site (which is still the main cause of cancer mortality). This knowledge is paramount for attaining more efficient diagnostic and chemotherapeutic approaches, aiming at an improved prognosis for oncology patients.

### Supplementary Information

Supplementary Information.

## Data Availability

The data that support the findings of this study are available from the corresponding author upon reasonable request.

## References

[1] Sung H (2021). Global cancer statistics 2020: GLOBOCAN estimates of incidence and mortality worldwide for 36 cancers in 185 countries. CA Cancer J. Clin..

[2] DeSantis CE (2019). Breast cancer statistics, 2019. CA Cancer J. Clin..

[3] Saraon PD, Drabovich AP, Jarvi KA, Diamandis EP (2014). Mechanisms of androgen-independent prostate cancer. EJIFCC..

[4] Dong L, Zieren RC, Xue W, Reijke TM, Pienta KJ (2019). Metastatic prostate cancer remains incurable, why?. Asian J. Urol..

[5] Brücher BLDM, Jamall IS (2019). Transition from normal to cancerous cell by precancerous niche (PCN) induced chronic cell-matrix stress. Open..

[6] Pavlova NN, Thompson CB (2016). The emerging hallmarks of cancer metabolism. Cell Metab..

[7] de Carvalho ALMB (2016). Chemotherapeutic response to cisplatin-like drugs in human breast cancer cells probed by vibrational microspectroscopy. Faraday Discuss..

[8] Lamego I (2017). Impact of the Pd_2_Spermine chelate on osteosarcoma metabolism: An NMR metabolomics study. J. Proteome Res..

[9] Baker MJ (2018). Clinical applications of infrared and Raman spectroscopy: State of play and future challenges. Analyst..

[10] Cross S, Jin YS, Rao J, Gimzewski JK (2007). Nanomechanical analysis of cells from cancer patients. Nat. Nanotech..

[11] Davies PC, Demetrius L, Tuszynski JA (2011). Cancer as a dynamical phase transition. Theor. Biol. Med. Model..

[12] Yoshii T, Geng Y, Peyton S, Mercurio AM, Rotello VM (2016). Biochemical and biomechanical drivers of cancer cell metastasis, drug response and nanomedicine. Drug Discov. Today..

[13] Pal SK, Zewail AH (2004). Dynamics of water in biological recognition. Chem. Rev..

[14] Sokolov AP, Roh JH, Mamontov E, García Sakai V (2008). Role of hydration water in dynamics of biological macromolecules. Chem. Phys..

[15] Frauenfelder HC, Chen G, Berendzen J, Young RD (2009). A unified model of protein dynamics. Acad. Sci..

[16] Luby-Phelps K (2013). The physical chemistry of cytoplasm and its influence on cell function: An update. Mol. Biol. Cell..

[17] Xu W (2012). Cell stiffness is a biomarker of the metastatic potential of ovarian cancer cells. PLoS ONE..

[18] Davidson R, Lauritzen A, Seneff S (2013). Biological water dynamics and entropy: A biophysical origin of cancer and other diseases. Entropy..

[19] Schiro G (2015). Translational diffusion of hydration water correlates with functional motions in folded and intrinsically disordered proteins. Nat. Commun..

[20] Ruggiero MR (2018). Evidence for the role of intracellular water lifetime as a tumour biomarker obtained by in vivo field-cycling relaxometry. Angew. Chem. Int. Ed. Engl..

[21] Rao C, Verma NC, Nandi CK (2019). Unveiling the hydrogen bonding network of intracellular water by fluorescence lifetime imaging microscopy. J. Phys. Chem. C..

[22] Jasnin M, Moulin M, Haertlein M, Zaccai G, Tehei M (2008). Down to atomic-scale intracellular water dynamics. EMBO Rep..

[23] Sakai VG, Arbe A (2009). Quasielastic neutron scattering in soft matter. Curr. Opin. Colloid Interface Sci..

[24] Zaccai G (2016). Neutrons describe ectoine effects on water H-bonding and hydration around a soluble protein and a cell membrane. Sci. Rep..

[25] Vural D (2017). Quasielastic neutron scattering in biology: Theory and applications. Biochim. Biophys. Acta Gen. Subj..

[26] Mamontov E (2017). Microscopic diffusion in hydrated encysted eggs of brine shrimp. Biochim. Biophys. Acta Gen. Subj..

[27] Marques MPM (2017). Intracellular water: An overlooked drug target? Cisplatin impact in cancer cells probed by neutrons. Phys. Chem. Chem. Phys..

[28] Seydel T (2018). Increased rate of solvent diffusion in a prototypical supramolecular gel measured on the picosecond timescale. Chem. Commun..

[29] Foglia F (2019). In vivo water dynamics in *Shewanella oneidensis* bacteria at high pressure. Sci. Rep..

[30] Grimaldo M, Roosen-Runge F, Zhang F, Schreiber F, Seydel T (2019). Dynamics of proteins in solution. Q. Rev. Biophys..

[31] Natali F (2019). Anomalous water dynamics in brain: A combined diffusion magnetic resonance imaging and neutron scattering investigation. J. R. Soc. Interface..

[32] Yu M (2020). One-dimensional nature of protein low-energy vibrations. Phys. Rev. Res..

[33] Li R (2020). Anomalous sub-diffusion of water in biosystems: From hydrated protein powders to concentrated protein solution to living cells. Struct. Dyn..

[34] Marques MPM (2020). Intracellular water as a mediator of anticancer drug action. Int. Rev. Phys. Chem..

[35] Marques MPM (2022). Water dynamics in human cancer and non-cancer tissues. Phys. Chem. Chem. Phys..

[36] de Carvalho ALMB (2019). Anticancer drug impact on DNA: A study by neutron spectroscopy coupled with synchrotron-based FTIR and EXAFS. Phys. Chem. Chem. Phys..

[37] Marques MPM (2019). chemotherapeutic targets in osteosarcoma: Insights from synchrotron-microFTIR and quasi-elastic neutron scattering. J. Phys. Chem. B..

[38] Marques MPM (2020). A new look into the mode of action of metal-based anticancer drugs. Molecules..

[39] Marques MPM (2020). Role of intracellular water in the normal-to-cancer transition in human cells-insights from quasi-elastic neutron scattering. Struct. Dyn..

[40] ISIS. http://www.isis.stfc.ac.uk/. Accessed Dec 2022.

[41] LET. https://www.isis.stfc.ac.uk/Pages/let.aspx. Accessed Dec 2022.

[42] Bewley RI, Taylor JW, Bennington SM (2011). LET, a cold neutron multi-disk chopper spectrometer at ISIS. Nucl. Instrum. Methods Phys. Res. A..

[43] Arnold O (2014). Mantid: Data analysis and visualization package for neutron scattering and μ SR experiments. Nucl. Instrum. Methods Phys. Res. A..

[44] Azuah RT (2009). DAVE: A comprehensive software suite for the reduction, visualization, and analysis of low energy neutron spectroscopic data. J. Res. Natl. Inst. Stand. Technol..

[45] Azrad M, Turgeon C, Demark-Wahnefried W (2013). Current evidence linking polyunsaturated Fatty acids with cancer risk and progression. Front. Oncol..

[46] Luo X (2017). Emerging roles of lipid metabolism in cancer metastasis. Mol. Cancer..

[47] Lyng FM (2019). Discrimination of breast cancer from benign tumours using Raman spectroscopy. PLoS ONE..

[48] Faria EC (2008). Measurement of elastic properties of prostate cancer cells using AFM. Analyst..

[49] Plodinec M (2012). The nanomechanical signature of breast cancer. Nat. Nanotechnol..

[50] Lekka M (2016). Discrimination between normal and cancerous cells using AFM. Bionanoscience..

[51] Lehuédé C, Dupuy F, Rabinovitch R, Jones RG, Siegel PM (2016). Metabolic plasticity as a determinant of tumor growth and metastasis. Cancer Res..

[52] Kreuzaler P, Panina Y, Segal J, Yuneva M (2020). Adapt and conquer: Metabolic flexibility in cancer growth, invasion and evasion. Mol. Metab..

[53] Mamontov E (2018). Microscopic diffusion processes measured in living planarians. Sci. Rep..

[54] Laage D, Hynes JTA (2006). Molecular jump mechanism of water reorientation. Science..

[55] Laage D (2009). Reinterpretation of the liquid water quasi-elastic neutron scattering spectra based on a nondiffusive jump reorientation mechanism. J. Phys. Chem. B..

[56] Laage D, Elsaesser T, Hynes JT (2017). Water dynamics in the hydration shells of biomolecules. Chem. Rev..

[57] Ziegler YS, Moresco JJ, Tu PG, Yates JR, Nardulli AM (2014). Plasma membrane proteomics of human breast cancer cell lines identifies potential targets for breast cancer diagnosis and treatment. PLoS ONE..

[58] Roy J, Wycislo KL, Pondenis H, Fan TM, Das A (2017). Comparative proteomic investigation of metastatic and non-metastatic osteosarcoma cells of human and canine origin. PLoS ONE.

[59] Mamede AP (2021). A new look into cancer: A review on the contribution of vibrational spectroscopy on early diagnosis and surgery guidance. Cancers.

[60] Santos IP, Martins CB, de Carvalho LAEB, Marques MPM, de Carvalho ALMB (2022). Who's who? Discrimination of human breast cancer cell lines by Raman and FTIR microspectroscopy. Cancers.

[61] Mamede AP (2022). Breast cancer or surrounding normal tissue? A successful discrimination by FTIR or Raman microspectroscopy. Analyst..

[62] Vanhove K (2019). The metabolic landscape of lung cancer: New insights in a disturbed glucose metabolism. Front. Oncol..

[63] Herath S (2021). The role of circulating biomarkers in lung cancer. Front. Oncol..

[64] Marjanovic ND (2020). Emergence of a high-plasticity cell state during lung cancer evolution. Cancer Cell..

[65] Vojtek M (2022). Pd_2_Spermine complex shows cancer selectivity and efficacy to inhibit growth of triple-negative breast tumors in mice. Biomedicines..

[66] Islam T, Resat H (2017). Quantitative investigation of MDA-MB-231 breast cancer cell motility: Dependence on epidermal growth factor concentration and its gradient. Mol. BioSyst..

[67] Chan JM (2022). Lineage plasticity in prostate cancer depends on JAK/STAT inflammatory signaling. Science..

[68] Evers MJT, Holt LJ, Alberti S, Mashaghi A (2021). Reciprocal regulation of cellular mechanics and metabolism. Nat. Metab..

[69] Bertolio R, Napoletano F, Del Sal G (2023). Dynamic links between mechanical forces and metabolism shape the tumor milieu. Curr. Opin. Cell Biol..

[70] Roshanzamir F (2022). Metastatic triple negative breast cancer adapts its metabolism to destination tissues while retaining key metabolic signatures. PNAS..

