# Supplementary material for: Cellular dynamics as a marker of normal-to-cancer transition in human cells
Source: Sci Rep. 2023 Nov 29;13:21079. doi: 10.1038/s41598-023-47649-w (PMC10687084; doi:10.1038/s41598-023-47649-w)
Supplement: Supplementary file 1 — Supplementary Information. [file 41598_2023_47649_MOESM1_ESM.pdf]

Supplementary Material

Cellular Dynamics as a Marker of Normal-to-Cancer Transition in Human Cells

M.P.M. Marques[a,b], A.L.M. Batista de Carvalho[a*], C.B. Martins[a,b], J.D. Silva[a,b], M. Sarter[c], V. García Sakai[c], J.R. Stewart[c] and L.A.E. Batista de Carvalho[a]

[a]University of Coimbra, Molecular Physical-Chemistry R&D Unit, Department of Chemistry, 3004-535 Coimbra, Portugal
[b]University of Coimbra, Department of Life Sciences, 3000-456 Coimbra, Portugal
[c]ISIS Facility, STFC Rutherford Appleton Laboratory, Chilton, Didcot, OX11 0QX, UK

Table of Contents

Materials and Methods

Chemicals and Cells Lines
Preparation of Cell Pellets for QENS Measurements
Quasi-elastic Neutron Scattering Fundamentals and Measurements
Quasi-elastic Neutron Scattering Data Analysis

Figures

Figure S1 – QENS profiles (at 310 K) for human lung cancer and lung healthy cells, fitted using three Lorentzians and one Delta functions, at some typical Q values for a 2.97 meV incident energy.

References

MATERIALS AND METHODS

Chemicals and Cell Lines

Bovine insulin 10 mg/mL, cholera toxin, DMEM-HG (Dulbecco's Modified Eagle Medium high glucose) culture medium, DMEM/F12 culture medium, EDTA (ethylenediaminetetraacetic acid, disodium salt dihydrate), F12 culture medium, hEGF (human epidermal growth factor), hydrocortisone, PBS (phosphate buffered saline), RPMI culture medium, sodium hydrogen carbonate and trypsin-EDTA (1×) were purchased from Sigma-Aldrich (UK). FBS (fetal bovine serum) and FHS (fetal horse serum) were obtained from Gibco (UK).

The human cell lines presently studied were purchased from: MDA-MB-231 (triple-negative breast carcinoma, breast cancer) – DSMZ, German Collection of Microorganisms and Cell Cultures GmbH (Leibniz Institute, Germany); MCF-12A (non-tumorigenic mammary immortalised cells, breast healthy) – ATCC, American Type Culture Collection (Manassas/Virginia, USA); PC-3 (androgen-independent prostatic adenocarcinoma, prostate cancer) and PNT-2 (normal prostate epithelial immortalised cells, prostate healthy) – ECCAC, European Collection of Cell Cultures (Salisbury, UK); A549 (non-small cell lung adenocarcinoma, lung cancer) and BEAS-2B (non-tumorigenic bronchial epithelium immortalised cells, lung healthy) – ATCC, American Type Culture Collection (Manassas/Virginia, USA).

Preparation of Cell Pellets for QENS Measurements

The cells were grown on-site (at the Biology laboratory of the ISIS Pulsed Neutron and Muon Source of the Rutherford Appleton Laboratory) [1], cultured as monolayers, at 37 °C in a humidified atmosphere of 5% CO_2. Breast cancer cell cultures were maintained in DMEM culture medium, supplemented with 10% (*v/v*) heat-inactivated FBS. For the breast healthy cell cultures DMEM/F12 medium was used, supplemented with 5% (*v/v*) heat-inactivated FHS, 20 ng/ml hEGF, 100 ng/ml cholera toxin, 0.01 mg/ml bovine insulin and 500 ng/ml hydrocortisone. The prostate cancer and prostate healthy cell lines were grown in RPMI medium supplemented with 10% (*v/v*) heat-inactivated FBS. The lung cancer cell line was cultured in RPMI medium, supplemented with 10% (*v/v*) heat-inactivated FBS and lung healthy cells were cultured in F12 medium, supplemented with 10% (*v/v*) heat-inactivated FBS. All cell lines were subcultured at 80% confluence, using a 0.05% trypsin-EDTA (1×) solution in PBS.

To avoid osmotic perturbations that might cause cell damage, all samples were prepared in deuterated isotonic media (phosphate saline buffer, PBS_{deut}). In order to eliminate the extracellular water, the cell pellets were washed with deuterated PBS by resuspension (1×) followed by centrifugation (at 195 × g) for 5 min, which was repeated for 15 min after removing the first supernatant. As previously verified by the authors [2], this process does not affect the integrity of the cells, and allows to observe only intracellular water and biomolecules in the densely packed cell pellets. Under these experimental conditions, the proportion of intracellular water was determined to be *ca.* 95% of the total water present, the extracellular water in the cell pellets being therefore than 5% [2,3].

It should be emphasised that even considering cold-triggered denaturation of some cellular components during spectral acquisition, the present study aims at a comparison between data obtained (at the same temperature) for different cell lines (healthy *versus* tumour and distinct cancer types) – hence, possible occasional cell-damaging effects should be identical across all samples and are expected to be cancelled out.

Quasi-elastic Neutron Scattering Fundamentals and Measurements

Quasi-elastic incoherent neutron scattering (QENS) typically analyses the incoherent scattering signal resulting from a variety of atomic motions ranging from fast vibrational and rotational localised modes, to slower diffusional modes, which take place at pico- to nanosecond timescales, and exploits the high sensitivity of neutrons to hydrogen (with a much larger incoherent scattering cross section compared to that of other elements). The QENS signal, the so-called dynamic structure factor, S(Q,ω), arises from a variety of dynamical processes (which fall in the spectrometer's time window) – from fast localised modes including vibrations and rotations to slower global translational motions. It is due to energy (ħω) and momentum (Q) exchanges between the neutrons and the atoms within a given spectrometer resolution, and is detected as a broadening about an elastic line of energy exchange ≈0. The measured signal can be expressed as:

$$S_{measured}(Q,\omega)=\exp\left(-\frac{\hbar\omega}{2kT}\right)R(Q,\omega)\otimes S(Q,\omega) \qquad (2)$$

where the exponential term is a detailed balance parameter, and R(Q,ω) is the instrument´s resolution function (experimentally obtained) which is convoluted with the scattering model (S(Q,ω)) that describes the dynamical behaviour of the sample. In biological samples (with a high hydrogen content) S(Q,ω) is dominated by the incoherent scattering of the hydrogen atoms (much larger than the coherent or incoherent scattering cross section of any other atom) and are approximated as the convolution of vibrational, rotational and translational components (taken as independent motions), according to the equation:

$$S_{inc}(Q,\omega)=S_{vib}(Q,\omega)\otimes S_{rot}(Q,\omega)\otimes S_{trans}(Q,\omega) \qquad (3)$$

Strictly in the elastic and quasielastic regions, the following expression applies:

$$S_{inc}(Q,\omega) = \exp(-Q^2\langle u^2\rangle)\left[A_0(Q)\delta(\omega) + \sum_l A_l(Q)L_l(Q,\omega)\right] \qquad (4)$$

where the exponential term is the Debye-Waller factor, $A_0(Q)\delta(\omega)$ is the elastic component assigned to motions slower than the longest observable time as defined by the spectrometer´s energy resolution, and the second term in the equation corresponds to the quasielastic contributions. The term $S_{inc}(Q,\omega)$ affords time/space information on the system probed – on the timescale of the dynamical processes (through the neutron energy transfer, ω) and on the spatial extent of these processes (*via* the momentum scattering transfer, Q). Since the QENS data in the time domain is represented by an exponential, it can be approximated in the energy domain by Lorentzian functions of different widths,

$$L(x) = \frac{1}{\pi}\frac{\Gamma}{\Gamma^2+\omega^2} \qquad (5)$$

Γ representing the full width at half-maximum (FWHM=2xHWHM (half-width at half-maximum)). These Lorentzian functions describe motions occurring on different timescales, the Q-dependency of Γ delivering detailed information for each dynamic component.

The QENS experiments were carried out at the ISIS Pulsed Neutron and Muon Source of the Rutherford Appleton Laboratory (UK), in the low-energy LET direct-geometry time-of-flight spectrometer [4,5], with simultaneous data acquisition at different incident neutron energies: 1.7, 2.97 and 6.42 meV, with energy resolutions (FWHM) equal to 27, 58 and 164 μeV, respectively. The data was recorded in a wide Q range, covering a -40º to 140º angular interval, within the energy transfer windows -1.36 – 1.36 meV (0.09 to 2.00 $Å^{-1}$), -2.38 – 2.38 meV (0.12 to 2.65 $Å^{-1}$) and -5.14 – 5.14 meV (0.18 to 3.89 $Å^{-1}$), respectively for each of the 1.7, 2.97 and 6.42 meV incident energies.

The samples, containing *ca.* 150 mg/1 cm^3 of cell pellet (*ca.* 5×10^8 cells), were wrapped in aluminium foil sachets (which filled the beam) and mounted in indium-sealed 0.1 mm-thick (3×5 cm) flat Al cans (the beam size at the sample being 2×4 cm), and were placed at 135° relative to the incident beam. The PBS_{deut} sample was placed in 0.4 mm-thick (3×5 cm) Al container to allow for a higher amount of scatterer in the beam (still keeping to a 10% scatterer to reduce the possibility of multiple scattering).

Experiments were performed at 310 K, to better represent the physiological conditions, the temperature being controlled to ±0.01 K using local resistance heating on the sample holders. QENS acquisition times were between 8 and 10 h (*ca.* 240 to 300 µA). Some experiments were run at low temperature (*ca.* 5 K) and the data used as a resolution function. A vanadium sample (a purely incoherent elastic scatterer) was also measured to define the instrument resolution and correct for detector efficiency. The QENS signal measured with the lowest incident neutron energy displays a significantly decreased intensity relative to the other two (higher) incident energies.

Quasi-elastic Neutron Scattering Data Analysis

The neutron data was reduced from raw time-of-flight signals into energy transfer using the MANTID program (version 3.4.0) [6]. QENS spectra were corrected for detector efficiency. Resolution functions were determined independently from calibration runs for vanadium.

Fitting of the QENS spectra was performed with DAVE (version 2.5, developed at the National Institute of Standards and Technology (NIST) Center for Neutron Research) [7]. The analysis was carried out sequentially, optimising different motions for each incident neutron energy: (i) starting by the results obtained at 2.97 meV – fitted with one Dirac Delta function (elastic component) and three Lorentzians (quasielastic contributions from the water in the cytoplasm and the biomolecules′ hydration layers, as well as from fast localised motions within the cell); (ii) following to the data measured at 1.70 meV – fitted with one Dirac Delta function (elastic component) and two Lorentzians (quasielastic contributions from cytoplasmic and hydration waters, the former optimised in the previous step and fixed in this step); (iii) finally fitting the results acquired at 6.42 meV – with one Dirac Delta function (elastic component) and two Lorentzians (quasielastic contributions from the fast dynamical processes and from cytoplasmic water, the latter optimised at 2.97 meV and fixed in this step). Overall, the systems were well represented by applying one Dirac Delta function (elastic component) and three Lorentzians (quasielastic contributions), plus an energy independent instrumental background (sloping) (Fig. S1). The choice for fixing one Lorentzian was based on mathematical accuracy (by comparison with fitting without a fixed Lorentzian). For the lowest (1.70 meV) and highest (6.42 meV) incident energies, three Lorentzians did not provide a good fit.

The dynamical contributions from hydration water and cytoplasmic water were more accurately obtained from the fittings at 1.7 and 2.97 meV incident energies, respectively, while the fastest motions within the cellular milieu were best fitted at 6.42 meV. FWHM values ($\Gamma_T(Q)$) were extracted from each of the Lorentzian functions. For the motions of cytoplasmic water, the translational diffusion coefficients (D_T) and reorientation times (τ_T^{jump}, the mean residence time of a water molecule in each possible location) (at temperature T) were obtained according to a non-diffusive jump reorientation model [8-10] that follows the equation:

$$\Gamma_T(Q) = \frac{D_T Q^2}{1+D_T Q^2 \tau_T^{jump}} \quad (6)$$

For hydration water, D_T and τ_T obeyed a Fickian model according to,

$$\Gamma_T = 2DQ^2 \quad (7)$$

FIGURES

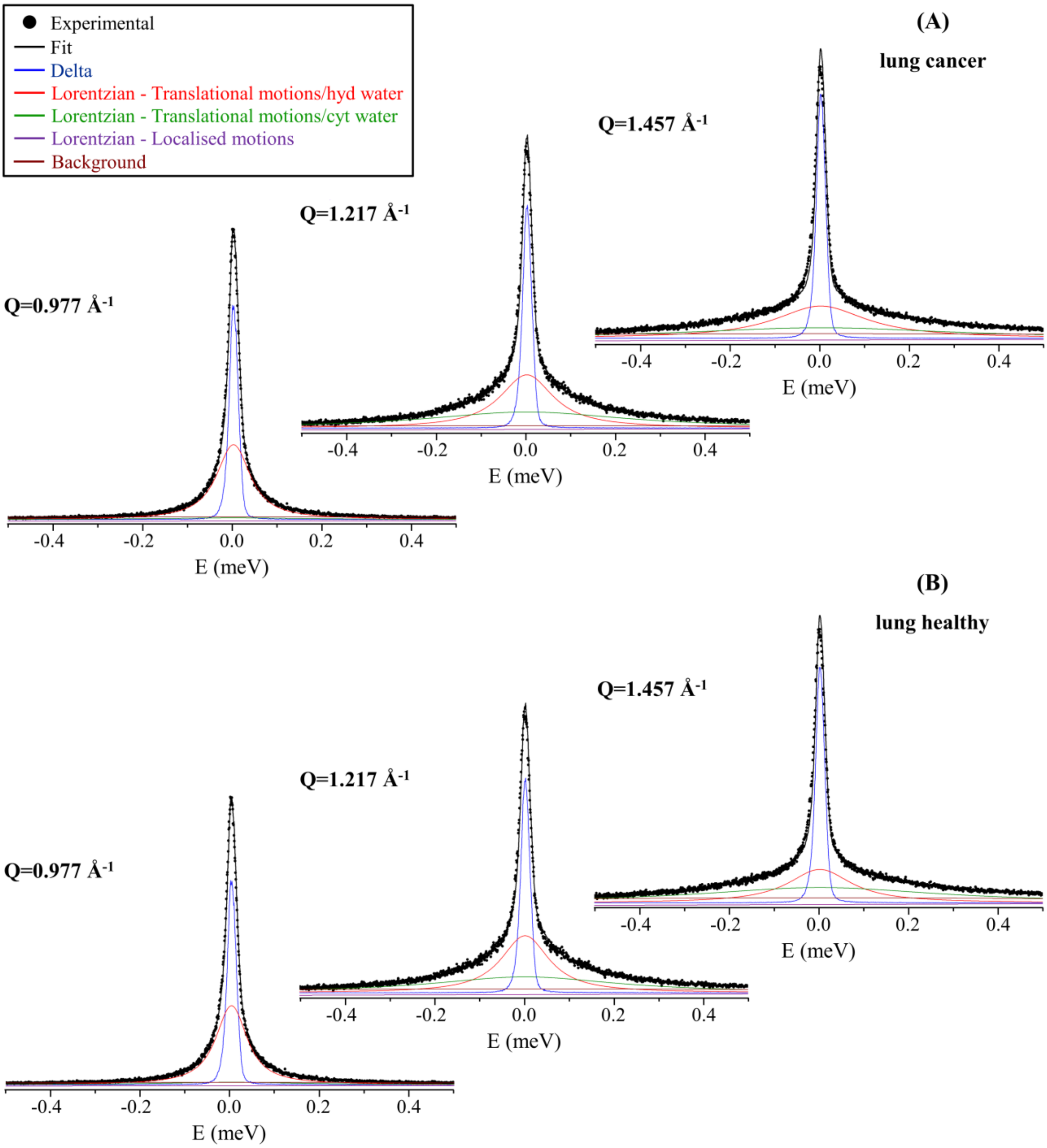

Figure S1 – QENS profiles (at 310 K) for human lung cancer and lung healthy cells, fitted using three Lorentzians and one Delta functions, at some typical Q values, for a 2.97 meV incident energy.

REFERENCES

[1] Biology Laboratories - ISIS Neutron and Muon Source "https://www.isis.stfc.ac.uk/Pages/Biology-Laboratories.aspx." (accessed March 2023).

[2] Marques, M. P. M. *et al*. Role of intracellular water in the normal-to-cancer transition in human cells-insights from quasi-elastic neutron scattering. *Struct Dyn*. **7(**5), 054701, https://doi.org/10.1063/4.0000021 (2020).

[3] Marques, M. P. M. *et* al. Chemotherapeutic Targets in Osteosarcoma: Insights from Synchrotron-MicroFTIR and Quasi-Elastic Neutron Scattering. *J Phys Chem B*. **123**(32), 6968-6979. https://doi.org/10.1021/acs.jpcb.9b05596 (2019).

[4] ISIS. "http://www.isis.stfc.ac.uk/." (accessed December 2022).

[5] Bewley, R. I., Taylor, J. W. & Bennington, S. M. LET, a cold neutron multi-disk chopper spectrometer at ISIS. *Nucl. Instrum. Methods Phys. Res. A*. **637(**1), 128-134. https://doi.org/10.1016/j.nima.2011.01.173 (2011).

[6] Arnold, O. *et al*. Mantid—Data analysis and visualization package for neutron scattering and μ SR experiments. *Nucl. Instrum. Methods Phys. Res. A*. **764**, 156-166. https://doi.org/10.1016/j.nima.2014.07.029 (2014).

[7] Azuah, R. T. *et al*. DAVE: a comprehensive software suite for the reduction, visualization, and analysis of low energy neutron spectroscopic data. *J. Res. Natl. Inst. Stand. Technol.* **114**(6), 341. https://doi.org/10.6028/jres.114.025 (2009).

[8] Laage, D. & Hynes, J. T. A. Molecular Jump Mechanism of Water Reorientation. *Science*. **311**(5762), 832-835. https://doi.org/10.1126/science.1122154 (2006).

[9] Laage, D. Reinterpretation of the liquid water quasi-elastic neutron scattering spectra based on a nondiffusive jump reorientation mechanism. *J Phys Chem B*. **113**(9), 2684-7. https://pubs.acs.org/doi/10.1021/jp900307n (2009).

[10] Laage, D., Elsaesser, T. & Hynes, J. T. Water Dynamics in the Hydration Shells of Biomolecules. *Chem Rev*. 117(16), 10694-10725. https://doi.org/10.1021/acs.chemrev.6b00765 (2017).
